# Impact of Positively Charged Backbone Modifications on the Properties of Spherical Nucleic Acids

**DOI:** 10.1002/smll.74920

**Published:** 2026-07-29

**Authors:** Katherine E. Bujold, Kacper Skakuj, John P. Cavaliere, Caroline D. Kusmierz, Chad A. Mirkin

**Affiliations:** ^1^ Department of Chemistry and International Institute for Nanotechnology Evanston Illinois USA

**Keywords:** cellular uptake, deoxyribonucleic guanidine (DNG), nucleic acid chemistry, oligonucleotide backbone modifications, spherical nucleic acids

## Abstract

Spherical nucleic acids (SNAs) are nanostructures consisting of densely packed, radially oriented oligonucleotides arranged on nanoparticle cores. They are highly relevant to nanomedicine due to their unique physicochemical and biological properties, including efficient uptake across a broad range of cell types (over 50). This uptake is mediated in part by interactions between the oligonucleotide shell and scavenger receptors, making the DNA charge and sequence critical factors governing cellular internalization pathways. Recent advances in deoxynucleic guanidines (DNGs), nucleic acid analogues featuring positively charged guanidinium backbones, provide an opportunity to systematically probe how DNA charge influences SNA uptake. In this work, we report the synthesis of SNAs incorporating DNG‐DNA chimeras and evaluate how DNG incorporation alters SNA properties and cellular internalization. We find that cellular uptake increases with the number of DNG inserts, but only when these modifications are displayed on the outer surface of the SNA architecture. Furthermore, receptor blocking experiments indicate that uptake depends not only on scavenger receptor interactions but also on broader cell membrane composition. These findings demonstrate that both structural design and chemical composition can be tuned to control SNA uptake, underscoring the promise of structural nanomedicine for directing SNA‐cell interactions and rationally designing next‐generation nanotherapeutics.

## Graphical abstract

A synthesis of spherical nucleic acids (SNAs) featuring cationic backbone modifications is presented, together with an analysis of their emerging properties. Introducing positively charged guanidinium groups near the SNA surface enhances cellular uptake and offers a pathway toward more precise control over SNA internalization.

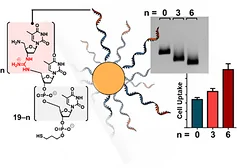

## 1 Introduction

The realization that oligonucleotide‐based architectures can exhibit emergent properties arising from their 3D structures has spurred the development of more effective drug delivery vehicles and stimuli‐responsive systems [1, 2, 3]. Within this context, an emerging paradigm, termed structural nanomedicine, emphasizes that nanoscale therapeutic efficacy is governed not only by composition but by the precise spatial arrangement, orientation, and valency of functional components within defined architectures [4, 5, 6]. This framework highlights the importance of establishing nanoscale structure‐activity relationships to rationally tune biological interactions and therapeutic performance. Within this landscape, spherical nucleic acids (SNAs), nanostructures composed of a nanoparticle core densely functionalized with radially oriented oligonucleotides, have emerged as particularly versatile architectures to address challenges in biology and medicine [1, 3, 7]. Both the core and oligonucleotide shell are synthetically addressable, enabling the creation of modular structures with programmable properties. SNAs are particularly attractive for biomedical applications as they are non‐toxic, exhibit efficient cellular uptake, show increased nuclease resistance, and display increased specificity towards binding with complementary sequences compared to free oligonucleotides [8]. These properties have established SNAs as a powerful and adaptable therapeutic platform with demonstrated efficacy in gene regulation [9, 10, 11, 12, 13, 14], drug delivery [15, 16], chemotherapy [17], and immunotherapy [18, 19, 20, 21, 22, 23].

Many of the desirable properties of SNAs, including rapid cellular uptake, arise from the dense oligonucleotide shell, which is recognized by class A scavenger receptors (SR‐A) on cell surfaces [24]. Because many cell types do not express SR‐A, efforts to modify the SNA platform to engage with cells more broadly or use alternate uptake mechanisms represent an attractive target for expanding the biomolecular recognition of SNAs and enabling new cellular uptake pathways and intracellular distribution [25, 26]. From a structural nanomedicine perspective, these modifications provide an opportunity to directly interrogate how nanoscale presentation of charge and functionality governs receptor engagement and cellular uptake pathways. Although numerous oligonucleotide modifications have been studied [27, 28, 29], cationic modifications of oligonucleotides have not been investigated because of the synthetic challenges involved [30, 31, 32, 33].

Studies of nanoparticles prepared with charged ligands suggest that modulating the surface charge of SNAs could improve their interactions with biomolecules and enhance bioavailability; however, contributions from the role, location, and number of cationic units necessary for optimal cellular uptake remain difficult to predict and achieve [34, 35, 36]. In structurally heterogeneous systems, these effects are often convoluted by polydispersity, making it difficult to isolate the role of charge as a defined structural variable. Alternatively, other groups have shown that complexing SNAs with cationic polymers (i.e., polyethylene imine) can enhance their cellular uptake and may alter their intracellular distribution [37]. However, electrostatically complexing a polydisperse polymer onto the SNA surface makes the structure more polydisperse and precludes careful structure‐function relationship analysis.

To address this challenge, guanidinium‐containing modifications were used because of their ability to promote the cellular uptake of arginine‐rich cell‐penetrating peptides, nanoparticles, proteins, polymers, and other conjugates in a large variety of cell types [38, 39, 40, 41, 42, 43, 44, 45, 46, 47]. More specifically, deoxynucleic guanidine (DNG) oligonucleotide modifications were used, in which the phosphate linkages of DNA were replaced with guanidinium moieties [30, 48, 49, 50]. The synthetic challenges associated with this backbone modification were addressed through the development of an automated route to the synthesis of DNG oligonucleotides, which demonstrated that linear DNG oligonucleotides readily enter cells compared to unmodified DNA. Thus, these DNGs offer a convenient way to study the impact of programmed charge modifications on the biological properties of SNAs [51, 52]. Here, incorporating cationic backbone modifications into structurally defined SNA architectures provides a route to precisely control both the magnitude and spatial presentation of charge, enabling a systematic investigation of how backbone chemistry, multivalency, and nanoscale organization collectively dictate biological function. We hypothesized that DNG modifications would be effective in enhancing the cellular uptake of SNAs, similar to what has been found for cell‐penetrating peptides, and enable new routes for cell entry.

## 2 Results and Discussion

### 2.1 Synthesis and Characterization of DNG‐DNA Chimeras

To test this hypothesis, SNAs containing varying numbers of cationic DNG inserts in different positions were synthesized (Figure 1A). For this work, poly‐thymidine (poly‐T) sequences were selected because of their low propensity for adopting secondary structures, and an overall oligonucleotide length of 20 bases was maintained across all constructs. Oligonucleotides bearing 1, 2, 3, 6 or 9 DNG modifications at either the 5' end or the 3' end, along with a 3' thiol modification to enable functionalization onto gold nanoparticles (AuNPs) were synthesized. The resulting SNAs contained DNG modifications either near the SNA outer surface or closer to the AuNP core, respectively (Table S1). The purity of the DNG‐DNA chimeric strands was assessed using MALDI‐TOF‐MS (Table S2) and polyacrylamide gel electrophoresis (PAGE) (Figure 1B; Figure S1).

**FIGURE 1:**
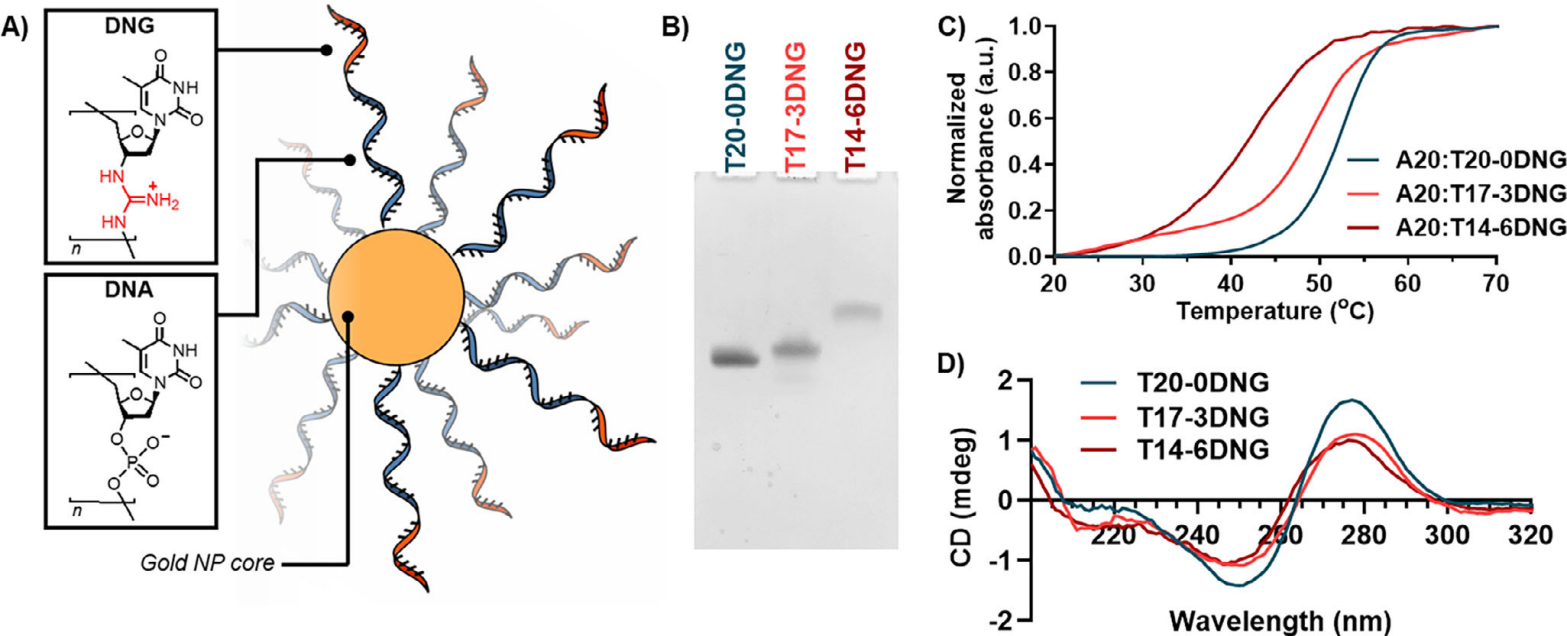
DNG SNA design and DNG‐DNA chimera characterization. (A) Schematic illustrating the structure and placement of DNG inserts on SNAs. (B) Electrophoretic mobility of DNG‐DNA chimeras on urea‐denaturing PAGE. (C) Melting curves of poly‐A:poly‐T duplexes with increasing numbers of DNG inserts. (D) Circular dichroism traces of DNG‐DNA chimeras with increasing numbers of DNG inserts.

The electrophoretic mobility of the DNG‐DNA chimeras depended on the number of DNG inserts and decreased with increasing numbers of DNG inserts due to the reduction in net negative charge (Figure 1B; Figure S1). DNG‐DNA chimeras containing 1, 2, 3, and 6 DNG inserts were soluble in water and exhibited expected DNA absorbance peaks at 260 nm. A nearly electroneutral 20‐mer chimera containing 9 DNG was also targeted; however, this sequence could not be purified without the use of strong surfactants because of its low aqueous solubility (Figures S1C and S2). Chimeras containing 1–6 DNG modifications hybridized with complementary poly‐adenine strands in 1x PBS exhibited lower melting temperatures compared to unmodified A20:T20 duplexes (1.2–1.9°C destabilization per insert), consistent with previous reports showing that DNG incorporation destabilizes duplex formation (Figure 1B; Figure S3 and Table S3) [53]. The circular dichroism spectra of these chimeras were similar to those for unmodified DNA in the single‐ and double‐stranded forms. Moreover, consistent with polythymidyl DNG oligomers, the circular dichroism spectra of the chimeras showed a reduction in intensity in both the 255 nm and 280 peaks and the emergence of a trough at 220 nm, suggesting a reduced ability to base‐stack and pre‐organize into helices (Figure 1C; Figure S4) [48]. Collectively, these results indicate that DNG inserts retain characteristic DNA features while destabilizing duplex formation. Interestingly, oligonucleotides containing a small number of DNG inserts (2 or 3) displayed temperature‐sensitive secondary structures even under urea‐denaturing PAGE conditions, consistent with electrostatic interactions that could be denatured at high temperatures but not using common chaotropic reagents (Figure S1A,B). These findings indicate that the introduction of DNG modifications within oligonucleotides reduces their binding affinity and helicity but also allows tunable electrostatic interactions to be explored.

### 2.2 Synthesis of DNG‐Modified SNAs

Thirteen‐nm citrate‐capped gold nanoparticles were functionalized with DNG‐DNA chimeras to form SNAs using three common methods: salt aging, freeze‐thaw, and pH‐assisted functionalization (Section S6.1; Figure S5), and oligonucleotide density was measured using fluorescence intercalation assays with Oligreen and agarose gel electrophoresis [54, 55, 56, 57]. The salt aging approach, which uses salt to screen the electrostatic repulsion between DNA strands at the AuNP surface, enables dense conjugation, but here the DNA loading achieved was dependent on the number of DNG units. Oligreen measurements revealed oligonucleotide loadings of 178, 162, 126, 117 and 232 strands/AuNP for unmodified, 1 DNG‐, 2 DNG‐, 3 DNG‐ and 6 DNG‐modified SNAs, respectively (Figure 2A). For the SNAs modified with 1–3 DNG, a decrease in loading is observed, which is likely due to electrostatic interactions between the guanidinium groups of DNGs and DNA phosphates, which promote the formation of bulky secondary structures that limit packing density. Notably, this trend reversed for chimeras containing 6 DNG units, which exhibited an increased loading of 232 strands/AuNP (Figure 2A), potentially due to stronger interactions between the cationic guanidinium groups and the AuNP surface that accelerate initial adsorption during salt aging. Together, these results indicate that DNG content modulates DNA loading during salt aging in a non‐linear manner.

**FIGURE 2:**
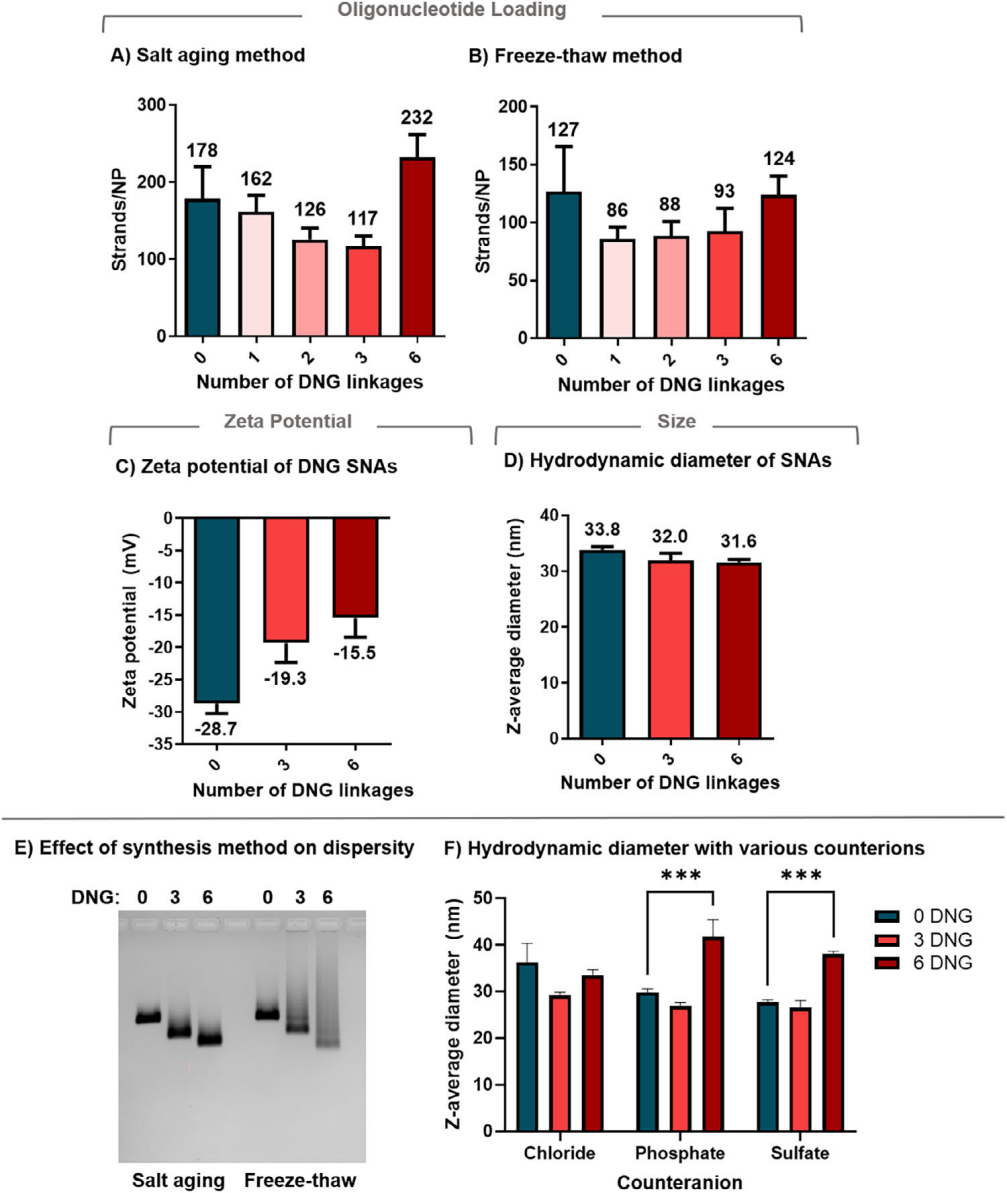
Characterization of DNG‐functionalized SNAs with DNG placement near the outer surface of the SNAs. (A) Oligonucleotide loading of salt‐aged SNAs with 0, 1, 2, 3 or 6 DNG inserts. (B) Oligonucleotide loading of freeze‐thawed SNAs with 0, 1, 2, 3 or 6 DNG inserts. (C) Zeta potential of DNG‐functionalized SNAs with 0, 3 or 6 DNG linkages from the salt aging method. (D) Hydrodynamic diameter of salt‐aged SNAs containing 0, 3 or 6 DNG linkages. (E) Electrophoretic mobility of DNG‐functionalized SNAs with 0, 3 or 6 DNG linkages made using different synthesis approaches. (F) Effect of counterion on the apparent size of DNG‐functionalized SNAs with 0, 3 or 6 DNG linkages made via the salt aging approach. Analysis of variance was conducted using a two‐way ANOVA with a Bonferroni multiple comparisons test. Asterisks denote statistical significance: *p < 0.05; **p < 0.01; ***p < 0.001. Mean loading densities, zeta potentials and hydrodynamic radii are indicated above each bar for panels (A–F). All results are reported as mean ± standard deviation.

In contrast, the freeze‐thaw method yielded lower and more uniform loading densities for DNG‐modified SNAs, highlighting key differences in functionalization behavior. This approach, which relies on transient concentration increases induced by ice crystal formation, produced SNAs with 86–93 strands/AuNPs for chimeras containing 1–3 DNG modifications (Figure 2B) [58]. Unlike salt aging, loading was relatively independent of DNG content at low DNA/AuNP ratios, suggesting reduced sensitivity to chimera composition. Although the 6 DNG construct achieved higher loading (124 strands/AuNP), this increase was less pronounced than that observed with salt aging (232 strands/AuNP; Figure 2A), likely due to the rapid kinetics of the freeze‐thaw process, which is completed within minutes. These results demonstrate that freeze‐thaw functionalization produces more consistent but overall lower loading densities than salt aging for DNG‐modified SNAs.

Beyond loading, the choice of functionalization method also impacts SNA physicochemical properties. Zeta potential measurements showed that increasing numbers of DNG inserts reduced surface charge in all cases due to the introduction of positively charged guanidinium groups, from −28.8 mV for unmodified SNAs to −15.5 mV for the 6 DNG construct made by salt aging (Figure 2C; Figure S6A,B). However, SNAs synthesized via salt aging were smaller and more monodisperse than those formed by freeze‐thaw, as evidenced by a decreasing hydrodynamic radii from 33.8 to 31.6 nm with increasing DNG content up to 6 inserts (Figure 2D; Figure S6C,D) This behavior is consistent with the slower, more controlled adsorption process in salt aging, which proceeds over days and favors thiol‐gold bonding over non‐specific guanidinium interactions. In contrast, freeze‐thaw led to aggregation, particularly at higher DNG contents, as indicated by gel smearing and increased hydrodynamic diameters of 66.8 nm for 3 DNG‐modified SNAs compared to 30.3 nm for unmodified SNAs (Figure 2E; Figures S6D and S7). This aggregation likely arises from reduced chemoselectivity under rapid (freeze‐thaw is completed within minutes), high‐concentration conditions, enabling guanidinium‐mediated cross‐interactions. Notably, aggregation was only observed when DNG units were positioned near the outer SNA surface, whereas constructs with DNGs closer to the AuNP core remained monodisperse (Figure S7). Overall, these observations demonstrate that salt aging produces structurally uniform DNG SNAs, whereas freeze‐thaw yields less controlled structures at high DNG content despite requiring a smaller excess of DNG‐DNA chimeras.

Finally, we investigated how DNG‐mediated interactions influence the structure of salt‐aged SNAs in aqueous media. The small hydrodynamic radii observed with increased DNG content suggest the formation of secondary structures stabilized by electrostatic and hydrogen‐bonding interactions involving guanidinium groups (Figure 2D). To probe this, SNAs were incubated with sodium salts containing different counterions (chloride, sulfate, and phosphate; Figure 2F). Relative to the unmodified SNAs, 6 DNG‐functionalized SNAs had increased hydrodynamic size in the presence of sulfate and phosphate, but not chloride, consistent with multivalent interactions between guanidinium groups and more highly charged anions. These findings support the hypothesis that guanidinium‐mediated interactions contribute to shell compaction and can be modulated through the ionic environment. Collectively, these results highlight the tunable and interactive nature of DNG‐functionalized SNAs prepared via salt aging and highlight their suitability for further study of their interactions with negatively charged biomolecules such as the anionic head groups of fatty acids, lipids, and glycosaminoglycans, which are relevant for cellular entry [38, 47].

### 2.3 Impact of DNG Modifications on the Cellular Uptake of SNAs

The electrostatic interactions and cationic charge of guanidiniums are widely considered to underlie their attractive biological properties, motivating their use as cell‐penetrating peptides and guanidinium‐based transporters [39, 40, 59, 60]. Guanidinium motifs promote cellular entry through both active and passive pathways even when conjugated to large macromolecular cargo [38, 59]. At first, unmodified SNAs and SNAs with 3 DNG modifications were assessed up to 400 nm by oligonucleotide using the AlamarBlue assay, which showed that they were non‐toxic to cells (Figure S8A). An RT‐qPCR assay also confirmed that DNG‐modified SNAs with 0, 1, 2, 3, or 6 DNG inserts did not adversely affect cellular transcriptional activity or overall cell health, consistent with them being non‐toxic to cells (Figure S8B,C).

To evaluate the effect of incorporating DNG inserts on SNA properties, cellular uptake was assessed using inductively coupled plasma mass spectrometry (ICP‐MS) and confocal microscopy with dye‐labeled DNG SNAs (section S7). Initially, DNG‐modified SNAs, with DNG modifications placed either near the outer surface of the SNAs or near the nanoparticle core, were incubated at 3 nm by AuNPs with HEK 293 cells for 5 h at 37°C and assessed using ICP‐MS. The results indicate that the presence of DNGs increased cellular uptake only when located on the outer surface of the SNA, and that this effect required at least 3 DNG modifications to be significant (Figure 3A; Figure S9). DNGs placed near the AuNP core had no measurable impact on cellular uptake, suggesting their availability on the surface of the SNA is key to their function. Moreover, this increase in uptake scaled with the number of DNG inserts and was observable in other cell lines. Incubation of DNG‐modified SNAs with 0, 3 or 6 DNG inserts (34 nm by dye, 1 nm by gold, 120 nm by oligonucleotide) in C166 cells for 5 h at 37°C also showed increases in cellular uptake with increasing DNG content by ICP‐MS and confocal microscopy (Figure 3B,C; Figure S10).

**FIGURE 3:**
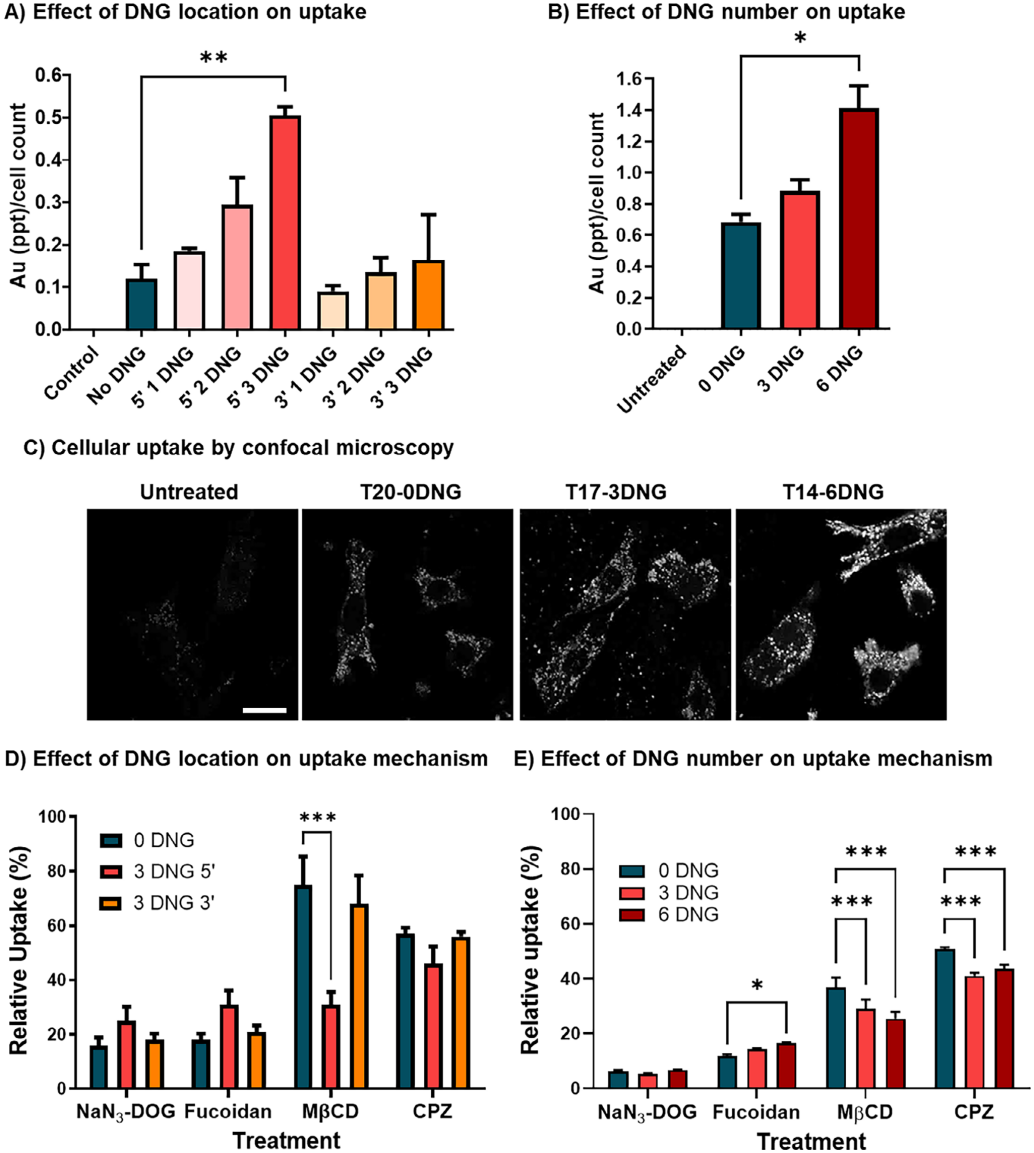
Cellular uptake of DNG‐modified SNAs. (A) Effect of DNG location on cellular uptake of SNAs (3 nm by AuNP, 400 nm by oligonucleotide) after a 5‐h incubation at 37°C in HEK 293 cells by ICP‐MS. (B) Effect of DNG number on cellular uptake of SNAs (1 nm by AuNP, 120 nm by oligonucleotide) after a 5‐h incubation at 37°C in C166 cells by ICP‐MS. (C) Cellular uptake of Cy5‐labeled DNG SNAs with up to 6 DNG inserts by confocal microscopy (34 nm by dye, 1 nm by gold, 120 nm by oligonucleotide) incubated for 5 h with C166 cells at 37°C. Scale bar represents 20 µm. The low levels of fluorescence in the untreated sample are attributed to autofluorescence. (D) Effect of DNG location on cellular uptake mechanism using a receptor blocking study. SNAs (3 nm by AuNP, 400 nm by oligonucleotide) were incubated with C166 cells for 1 h following pre‐treatment with drug for 0.5 h at 37°C. Cellular uptake in the presence of inhibitor is measured relative to untreated samples. (E) Effect of DNG number on cellular uptake mechanism using a receptor blocking study. SNAs (3 nm by AuNP, 400 nm by oligonucleotide) were incubated with C166 cells for 1 h following pre‐treatment with drug for 0.5 h at 37°C. Cellular uptake in the presence of inhibitor is measured relative to untreated samples. Asterisks denote statistical significance: ^*^ *p* < 0.05; ^**^ *p* < 0.01; ^***^ *p* < 0.001.

To understand the origin of the enhanced cellular uptake, the uptake mechanism of DNG chimera‐functionalized SNAs was investigated through a receptor blocking study [24]. C166 cells were pretreated with an excess of inhibitor for 0.5 h to competitively inhibit specific receptor‐mediated internalization pathways prior to SNA incubation (3 nm by AuNP, 400 nm by oligonucleotide) for 1 h. The uptake of SNAs was quantified in the presence of each blocker relative to untreated samples to elucidate the contribution of specific mechanisms of SNA internalization. The effect of DNG location and number of inserts was evaluated by comparing uptake profiles relative to those of unmodified SNAs.

First, the reliance of DNG‐modified SNAs on active transport processes was measured by incubating cells with sodium azide and 2‐deoxyglucose (NaN_3_‐DOG) prior to adding the SNAs, thereby depleting intracellular ATP and suppressing active transport mechanisms for cellular uptake. Under these conditions, the differences in uptake between the DNG‐modified and unmodified SNAs were marginal, indicating that the DNG inserts did not substantially increase cellular uptake through passive processes compared to unmodified SNAs (Figure 3D,E). The reliance of DNG SNAs on energy‐dependent pathways for uptake was also evidenced by confocal microscopy, where uptake of all SNAs (7.5 nm by dye, 2.5 nm by gold, 225 nm by oligonucleotide) is extensive at 37^°^C but greatly reduced at 4^°^C when incubated with HEK 293 cells for 2 h (Figure S9). Interactions between surface‐functionalized DNG‐modified SNAs and the cell membrane were evident as fluorescent bands around the edge of cells at 4°C, while accumulation in cytosolic compartments was observed at 37°C, suggesting that active transport is required for cellular uptake.

Next, fucoidan was used as a specific blocker of SR‐A receptors to determine if DNG‐functionalized SNAs undergo SR‐A‐mediated endocytosis, similar to conventional SNAs [24]. For all constructs tested, SR‐A‐mediated uptake remained prevalent as evidenced by their low relative cell uptake in the presence of fucoidan. Interestingly, only the surface‐functionalized 6 DNG SNAs showed a significantly decreased reliance on this uptake mechanism when compared to unmodified SNAs or other DNG‐modified SNAs as evidenced by their higher relative cellular uptake remaining after treatment with the blocker. This suggests that a sufficiently high density of guanidinium groups is required to decrease their reliance on this cell entry mechanism (Figure 3D,E).

Interestingly, blocking lipid raft‐ and caveolae‐mediated endocytosis with methyl‐β‐cyclodextrin (MβCD) led to significant changes in the magnitude of cell uptake of DNG SNAs. MβCD disrupts cell membrane composition by extracting cholesterol and fatty acids, which then inhibits cell uptake pathways dependent on membrane cholesterol. The presence of this blocker greatly reduced the uptake of SNAs with peripheral DNGs as opposed to unmodified SNAs and SNAs with DNG closer to the core, suggesting that they more highly depend on lipid raft‐ and caveolae‐mediated endocytosis than unmodified SNAs for cell entry (Figure 3D,E). This result underscores the importance of membrane composition for DNG SNA uptake and is in line with other reported systems relying on guanidiniums for uptake [40].

Finally, blocking clathrin‐mediated uptake with chlorpromazine (CPZ) yielded similar results, although the effect was less pronounced, suggesting DNG inserts can help promote this uptake pathway for SNAs to a small extent compared to unmodified SNAs (Figure 3D,E). However, the attenuated impact of CPZ compared to MβCD indicates that clathrin‐mediated uptake is not the dominant entry pathway for DNG‐modified SNAs. Importantly, the differential effects observed across inhibitors highlight that DNG placement and surface density not only influence overall uptake magnitude but also bias SNAs toward distinct endocytic pathways. Collectively, these results suggest that modifications to the phosphate backbone can be a versatile route for accessing multiple energy‐dependent cell entry mechanisms with SNAs.

## 3 Conclusion

In conclusion, we investigated the impact of incorporating increasing numbers of DNG backbone modifications on the synthesis and properties of SNAs. In contrast to modifications that retain the negative charge of DNA, the incorporation of positively charged groups has a profound impact on the synthesis, physical properties, and dispersity of the resulting SNAs, but it does not preclude the synthesis of monodisperse SNAs using the salt aging approach. Interestingly, the guanidinium groups of the DNG modifications retain their counterion binding properties, and even a few modifications result in increased cellular uptake. Investigations into the uptake mechanism of DNG‐modified SNAs illustrate their reduced reliance on SR‐A for uptake when densely functionalized and their increased dependence on cell entry mechanisms reliant on the presence of membrane cholesterol, such as lipid raft‐ and caveolae‐mediated endocytosis. DNG SNAs also show an increased dependence on clathrin‐mediated endocytosis. This is the first example of an alteration in the uptake mechanism of SNAs without the use of external agents. Collectively, these findings indicate that backbone modifications represent a modular strategy to tune the uptake mechanism and physical properties of SNAs, highlighting how precise structural control at the molecular level can be leveraged to direct nanoscale behavior in a manner consistent with emerging principles in structural nanomedicine.

## Funding

Research reported in this publication was supported by the National Cancer Institute of the National Institutes of Health awards R01CA257926 and R01CA275430, and the National Institute of Diabetes and Digestive and Kidney Diseases of the National Institutes of Health award U54DK137516. The content is solely the responsibility of the authors and does not necessarily represent the official views of the National Institutes of Health. This material is also based upon work supported by the National Science Foundation grant DMR‐2428112, Edgar H. Bachrach through the Bachrach Family Foundation, the Pat & Shirley Ryan Family Research Acceleration Fund, and the Robert H. Lurie Comprehensive Cancer Center. K. E. B. was supported by a Banting Fellowship from the Government of Canada.

## Conflicts of Interest

The authors declare no conflicts of interest.

## Supporting information




**Supporting File**: smll74920‐sup‐0001‐SuppMat.docx.

## Data Availability

The data that support the findings of this study are available in the supplementary material of this article.
