# Supplementary material for: Impact of Positively Charged Backbone Modifications on the Properties of Spherical Nucleic Acids
Source: Small. 2026 Jul 29;22(55):e74920. doi: 10.1002/smll.74920 (PMC13523125; doi:10.1002/smll.74920)
Supplement: Supplementary file 1 [file SMLL-22-e74920-s001.docx]

Supporting Information

Impact of Positively Charged Backbone Modifications on the Properties of Spherical Nucleic Acids

Katherine E. Bujold^‡^, Kacper Skakuj^‡^, John P. Cavaliere, Caroline D. Kusmierz, Chad A. Mirkin*

Department of Chemistry and International Institute for Nanotechnology, 2145 Sheridan Road, Evanston IL, 60208, United States

E-mail: [chadnano@northwestern.edu](mailto:chadnano@northwestern.edu)

**Table of Contents**

[1.	Materials	2](#_Toc234155275)

[2.	Instrumentation	2](#_Toc234155276)

[3.	Monomer synthesis	3](#_Toc234155277)

[3.1	Synthesis of the initiating (**I5**) and propagating (**P6**) units	3](#_Toc234155278)

[3.2	Synthesis of the capping unit (**C3**)	3](#_Toc234155279)

[4.	Oligonucleotide synthesis	8](#_Toc234155280)

[4.1	Standard oligonucleotide synthesis using phosphoramidite chemistry	8](#_Toc234155281)

[4.2	Coupling of the initiating unit (**I5**)	8](#_Toc234155282)

[4.3	DNG couplings using the mercuric chloride/thiophenol approach	8](#_Toc234155283)

[4.4	DNG couplings using the iodine/TMP approach	9](#_Toc234155284)

[4.5	Deprotection and work-up of oligonucleotides	9](#_Toc234155285)

[4.6	Oligonucleotide sequences	9](#_Toc234155286)

[5.	Characterization of DNG-DNA chimeras	11](#_Toc234155287)

[5.1	MALDI-TOF-MS characterization of DNG-DNA chimeras	11](#_Toc234155288)

[5.2	Characterization of DNG-DNA chimeras using gel electrophoresis	11](#_Toc234155289)

[5.3	Melting temperature by UV-visible spectroscopy	12](#_Toc234155290)

[5.4	Secondary structure by circular dichroism	13](#_Toc234155291)

[6.	Synthesis and characterization of DNG spherical nucleic acids (SNAs)	14](#_Toc234155292)

[6.1	Synthesis of SNAs	14](#_Toc234155293)

[6.2	Zeta potential and dynamic light scattering (DLS)	15](#_Toc234155294)

[6.3	Electrophoresis on agarose	16](#_Toc234155295)

[7.	Impact of DNG-modifications on the cellular uptake of SNAs	17](#_Toc234155296)

[7.1	Cell viability of DNG SNAs using the AlamarBlue assay	17](#_Toc234155297)

[7.2	eGFP expression and cell viability using RT-qPCR	17](#_Toc234155298)

[7.3	Cellular uptake by ICP-MS	18](#_Toc234155299)

[7.4	Cellular uptake by confocal microscopy	19](#_Toc234155300)

[7.5	Drug challenge by flow cytometry	20](#_Toc234155301)

[7.6	Statistical analysis	21](#_Toc234155302)

[8.	References	21](#_Toc234155303)

# 1. Materials

All reagents used in monomer synthesis and deoxyribonucleic guanidine (DNG) couplings, 30% aqueous ammonium hydroxide, 40% aqueous methylamine, 1 M triethylammonium acetate (TEAA) buffer at pH 7, Tween-20, sodium chloride and human serum were acquired from Sigma-Aldrich and used without further purification. Reagents for oligonucleotide synthesis, Cyanine 5 (Cy5) phosphoramidite (10-5915-90), 5'-thiol-modifier C6 S-S (10-1936-90) and 3'-thiol-modifier C3 S-S controlled pore glass (CPG) solid support beads (20-2933-10) were purchased from Glen Research. 1 M tris(hydroxymethyl)aminomethane (Tris) buffer pH 8, Dulbecco’s modified Eagle media (DMEM), Opti-MEM reduced serum media, 0.25% trypsin-EDTA, penicillin/streptomycin (100×), fetal bovine serum (FBS), trypan blue, Hoechst 33342 solution, 8-well chambered coverglass were obtained from ThermoFisher Scientific. HPLC-grade acetonitrile, gel electrophoresis supplies and buffers, Quant-iT OliGreen ssDNA reagent, disposable cuvettes for dynamic light scattering (Malvern Panalytical, ZEN0040) and zeta potential (Malvern Panalytical, DTS1070), phosphate-buffered saline (PBS), cell culture plates, hydrochloric acid (HCl), nitric acid (HNO_3_), 1000 ppm gold standard solution were purchased from Fisher Scientific. 2',6'-dihydroxyacetophenone (DHAP) was acquired from TCI Chemicals. Citrate-capped 13-nm gold nanoparticles were synthesized in-house according to previously reported protocols.^1^ 1× Tris-glycine-SDS buffer is composed of 25 mM Tris, 192 mM glycine and 0.1% sodium dodecyl sulfate (SDS) at a pH of 8.6. 1× Tris-boric acid-EDTA (TBE) buffer is composed of 89 mM Tris, 89 mM boric acid and 2 mM EDTA at a pH of 8.3.

# 2. Instrumentation

All NMR spectra were recorded on a Bruker Avance III instrument (500 MHz) using the solvent as internal standard. Electrospray ionization mass spectrometry (ESI–MS) data was acquired through direct injection on a Bruker amaZon SL instrument. Automated oligonucleotide synthesis was performed on either a MerMade 12 oligonucleotide synthesizer (BioAutomation) or an ABI 394 (Applied Biosystems) using standard phosphoramidite chemistry or the iodine-2,2,6,6-tetramethylpiperidine (TMP) strategy described previously.^2,3^ HPLC purifications were performed on either a Varian ProStar or a Shimadzu Prominence instrument equipped with a UV detector and C4 or C18 reverse-phase columns. Matrix-assisted laser desorption/ionization–time-of-flight mass spectrometry (MALDI-TOF-MS) was conducted on either a Bruker AutoFlex III or a Bruker Rapiflex using DHAP as the matrix. Polyacrylamide gel electrophoresis (PAGE) was carried on either a mini-PROTEAN system from Bio-Rad or a Hoefer 600 box from Fisher Scientific. Agarose electrophoresis was performed on Thermo Scientific Owl Easy Cast B2 mini gel electrophoresis systems from Fisher Scientific. Gels were scanned on a ChemiDoc MP imager from Bio-Rad or an Amersham Typhoon imaging system from GE Life Sciences. UV-visible measurements were performed on a Cary 100 or 300 UV-Visible (UV-Vis) spectrophotometer from Agilent. Fluorescence measurements were recorded on a Cytation 5 microplate reader (BioTek). Dynamic light scattering and zeta potential measurements were performed on a Malvern Zetasizer Nano instrument. Circular dichroism spectra were acquired on a Jasco J-1700 spectropolarimeter. Cells were counted on a Countess II Automated Cell Counter from Thermo Fisher Scientific. Inductively coupled plasma mass spectrometry (ICP-MS) was carried out on a Thermo iCAP Q instrument with 2% HCl/2% HNO_3_ as the matrix. Confocal microscopy was performed on a Zeiss LSM 800 confocal laser scanning microscope equipped with 405, 488 and 633 nm laser lines using constant laser power and PMT settings with a 63×/1.40 oil immersion objective. Flow cytometry analyses were conducted on a Guava easyCyte 8HT (Millipore Sigma) equipped with 488 and 642 nm laser lines by first identifying cells using forward and side scattering intensities, then reading fluorescence intensities at a constant 642 nm excitation.

# 3. Monomer synthesis

## 3.1 Synthesis of the initiating (**I5**) and propagating (**P6**) units

The initiating unit (**I5**) and the propagating unit (**P6**) were synthesized from thymidine based on reported procedures from Bruice^4,5^ and others^6-9^ applying modifications previously described by the authors.^2,3^

## 3.2 Synthesis of the capping unit (**C3**)

The capping unit (**C3**) was synthesized based on previous reports from Bruice^4,5^ and others^6-9^ with modifications by the authors as outlined in Scheme S1 in 38% overall yield. Azidothymidine was selected as precursor instead of thymidine to reduce the number of synthetic steps.

Scheme S1. Synthesis of the capping unit (C3). (a) 4-Monomethoxytrityl chloride (MMTr-Cl), 25% triethylamine (TEA) in dichloromethane (DCM), 16 h, 78%; (b) H_2_, Palladium over carbon (Pd/C), methanol (MeOH), 72 h, 59%; (c) Fluorenylmethoxycarbonyl isothiocyanate (Fmoc-NCS), DCM, 16 h, 81%.

3.2.1 ***5'-O-monomethoxytrityl-3'-azido-3',5'-deoxythymidine (C1)***

Dissolved azidothymidine (5.0 g, 18.6 mmol) in 25% TEA in DCM (50 mL) under nitrogen, then added MMTr-Cl (5.74 g, 18.6 mmol, 1 equiv.), pre-dissolved in 25 mL of the same solvent, dropwise over 10 minutes. The mixture was allowed to stir overnight prior to evaporation of the solvent. The resulting solid was washed with diethyl ether, yielding **C1** as a white foam. (78% yield)

**^1^H NMR** (400 MHz, Methanol-*d_4_*) δ 7.69 (d, *J* = 1.3 Hz, 1H), 7.47 (ddd, *J* = 8.2, 2.5, 1.3 Hz, 4H), 7.38 – 7.24 (m, 8H), 6.94 – 6.88 (m, 2H), 6.21 (t, *J* = 6.4 Hz, 1H), 4.55 – 4.49 (m, 1H), 4.02 – 3.96 (m, 1H), 3.81 (s, 3H), 3.53 – 3.47 (m, 1H), 3.39 (dd, *J* = 10.8, 3.4 Hz, 1H), 2.60 – 2.51 (m, 1H), 2.47 – 2.39 (m, 1H), 1.51 (d, *J* = 1.2 Hz, 3H).

3.2.2 ***5'-O-monomethoxytrityl-3'-amino-3',5'-deoxythymidine (C2)***

Dissolved **C1** (7.9 g, 14.5 mmol) in degassed methanol under nitrogen. A slurry of 10% Pd/C (400 mg) in degassed methanol was added to the solution, which was then exposed to a positive pressure of hydrogen for 72 h with stirring. The catalyst was removed by filtration over a pad of Celite using methanol as rinse and the solvent was evaporated. The crude was purified on silica, eluting unreacted and side products with ethyl acetate and methanol, prior to eluting the product with 4% aqueous ammonia in methanol. Yields **C2** as a yellowish foam. (59% yield)

**^1^H NMR** (400 MHz, Methanol-*d_4_*) δ 7.69 (d, *J* = 1.4 Hz, 1H), 7.47 (dt, *J* = 8.1, 1.4 Hz, 4H), 7.35 – 7.24 (m, 8H), 6.93 – 6.85 (m, 2H), 6.28 (dd, *J* = 6.8, 5.3 Hz, 1H), 3.87 (dt, *J* = 6.3, 3.4 Hz, 1H), 3.80 (d, *J* = 1.1 Hz, 3H), 3.76 – 3.66 (m, 1H), 3.50 (dd, *J* = 10.6, 2.8 Hz, 1H), 3.42 (dd, *J* = 10.6, 3.9 Hz, 1H), 2.40 (ddd, *J* = 13.2, 7.6, 5.3 Hz, 1H), 2.25 (dt, *J* = 13.5, 6.6 Hz, 1H), 1.46 (d, *J* = 1.2 Hz, 3H).

3.2.3 ***N-fluorenylmethoxycarbonyl-N'-[(3',5'-deoxythymidin-3'-yl)-5'-O-monomethoxytrityl] thiourea***

Dissolved **C2** (1.5 g, 2.9 mmol) in DCM with stirring under nitrogen. Fmoc-NCS (1.2 g, 4.3 mmol, 1.5 equiv.) was added directly to the solution and the reaction was allowed to stir overnight. The solvent was evaporated, and the crude was purified on silica using a DCM to ethyl acetate gradient. Affords **C3** as a yellowish foam. (81% yield)

**ESI–MS** (m/z): [M + Na]^+^ calculated for C_46_H_42_N_4_NaO_7_S: 817.27; found: 817.30; [M + Cl]^-^ calculated for C_46_H_42_ClN_4_O_7_S: 829.25; found: 829.24.

**^1^H NMR** (400 MHz, Methanol-*d_4_*) δ 7.83 (d, *J* = 7.6 Hz, 2H), 7.76 – 7.63 (m, 3H), 7.59 – 7.09 (m, 16H), 6.93 – 6.81 (m, 2H), 6.28 – 6.23 (m, 1H), 5.37 (q, *J* = 6.6 Hz, 1H), 4.56 – 4.45 (m, 2H), 4.31 (t, *J* = 6.8 Hz, 1H), 4.18 – 4.13 (m, 1H), 3.77 (s, 3H), 3.55 – 3.43 (m, 2H), 2.71 (dt, *J* = 13.8, 7.3 Hz, 1H), 2.45 (dt, *J* = 13.5, 6.6 Hz, 1H), 1.48 (d, *J* = 1.2 Hz, 3H).

# 4. Oligonucleotide synthesis

## 4.1 Standard oligonucleotide synthesis using phosphoramidite chemistry

All oligonucleotides with phosphate backbones were synthesized in-house on either a MerMade 12 or an ABI 394 automated oligonucleotide synthesizer using standard phosphoramidite chemistry protocols and standard reagents at the 1, 5 or 10 μmol scale on 3'-thiol-modifier C3 S-S CPG (Glen Research, 20-2933-10).^10^ Cy5 phosphoramidite was coupled by hand according to the procedure outlined in section 4.2.

## 4.2 Coupling of the initiating unit (**I5**)

Initiating monomer (**I5**) was added onto the 5' hydroxyl group of the growing strand using standard phosphoramidite chemistry. Couplings were performed by hand at the 1, 5 or 10 μmol scale in a glove box, to ensure an inert atmosphere and minimize losses of monomer due to priming of the lines on the synthesizer. Briefly, CPG columns were de-tritylated at the 5' end with deblock (3% dichloroacetic acid in DCM) over 5 min, rinsed with acetonitrile and dried before being brought into the glove box. Equal volumes of activator (0.25 M 5-benzylthio-1*H*-tetrazole in anhydrous acetonitrile) and **I5** solution at 0.1 M in DCM were then added onto the CPG beads such that the ratio of **I5** to growing DNA strands was 20:1. The coupling reaction was allowed to proceed for 15 min at room temperature with regular mixing. Once completed, the coupling solution was removed, and the CPG beads were washed with anhydrous acetonitrile. Oxidation of the phosphoramidite linkage was performed using CSO oxidizer (Glen Research, 40-4632-52), due to its compatibility with glove box environments, over 5 min followed by washing with anhydrous acetonitrile. Capping was performed outside the glove box using equal volumes of Cap A (80% tetrahydrofuran (THF)/10% pyridine/10% acetic anhydride) and Cap B (16% 1-methylimidazole in THF). Once the coupling cycle was completed, CPG beads were washed with acetonitrile, dried, and stored at -20 °C.

## 4.3 DNG couplings using the mercuric chloride/thiophenol approach

All DNG couplings procedures using mercuric chloride and thiophenol were adapted from reports by the Bruice group^4,5^ with minor modifications. DNA strands terminated with **I5** were deprotected using deblock to remove the terminal monomethoxy trityl (MMTr) protection group over 5 min, then washed with acetonitrile and dried, after which the deprotected CPG beads were brought into the glove box. Couplings were performed at the 5 μmol scale by adding 1 mL of monomer (either **P6** or **C3**, 75 mM in *N*,*N­*-dimethylformamide (DMF), 15 equivalents to growing strand) and then simultaneously adding 0.5 mL of each mercuric chloride (HgCl_2_, 100 mM in DMF) and TEA (200 mM in DMF). The coupling reaction was thoroughly mixed and allowed to proceed for 1.5 h at room temperature after which it was removed from the beads. Typically, the reaction mixture initially turned yellow, then dark gray over time with the apparition of a precipitate. The precipitate, as well as gray aspect of the CPG beads were removed using 20% thiophenol in DMF, followed by copious washing with DMF, after which the columns were removed from the glove box. Capping was performed using equal volumes of Bruice’s Cap A (100 mM acetic anhydride in DMF) and Bruice’s Cap B (200 mM TEA in DMF) for 5 min, followed by successive washes with DMF, MeOH and DMF. These DNG coupling cycles were repeated to add DNG linkages as needed.

## 4.4 DNG couplings using the iodine/TMP approach

All DNG couplings using the iodine/TMP approach were performed at the 1 μmol scale according to procedures reported by the authors on a MerMade 12 automated oligonucleotide synthesizer.^2,3^ Briefly, CPG beads containing different lengths of polythymidine were initially functionalized with **I5** as described in section 4.2 and loaded into the synthesizer. Strands were de-tritylated at the beginning of each coupling cycle, followed by acetonitrile, DMF and acetonitrile washes. Coupling was performed by adding **P6** or **C3** (25 mM in 1:1 ACN:DCM) and DNG activator (50 mM iodine, 150 mM TMP) at a 3:1 ratio and incubating for 10 min with 2 pulses. Coupling was repeated, followed by an acetonitrile rinse, then the oligonucleotide was capped with standard reagents and rinsed with acetonitrile. DNG coupling cycles were repeated as needed to produce mixed DNG–DNA chimeras.

## 4.5 Deprotection and work-up of oligonucleotides

All oligonucleotides were cleaved from CPG beads and deprotected at 55 °C in either 30% aqueous ammonium hydroxide overnight or 1:1 30% aqueous ammonium hydroxide: 40% methylamine for 1 h. After deprotection, 1 M Tris buffer pH 8 was added to a final concentration of approximately 100 mM to prevent loss of the terminal MMTr group in the case of DNG-terminated strands, and the oligonucleotide was concentrated under a nitrogen stream. Once the ammonia and methylamine were evaporated, the oligonucleotide was filtered through a 0.2 μm syringe filter and purified by HPLC using the trityl-ON approach, accomplished by running a 50-min linear gradient from 100 mM TEAA buffer to acetonitrile. Interestingly, later eluting peaks appeared as the number of DNG inserts increased. We hypothesize these arise from the electrostatic assembly of these chimeras, which become more prominent as the number of DNG inserts is increased. In the case of T11-9DNG, no product peak could be detected by HPLC.

Product peaks were lyophilized and resuspended in 20% acetic acid for 1 h to remove the terminal trityl group, which was subsequently extracted using ethyl acetate. Purified oligonucleotides were lyophilized, resuspended in ultrapure water, filtered to 0.2 μm and quantified by UV-Visible spectroscopy using their absorbance at 260 nm and extinction coefficients calculated for polythymidine strands of equal length using the OligoAnalyzer tool (IDT Technologies) to determine their molar concentrations. Successful synthesis and purification were confirmed with MALDI-TOF-MS, using DHAP as the matrix, and denaturing PAGE. Oligonucleotides were stored at 4 °C or -20 °C until further use.

## 4.6 Oligonucleotide sequences

All oligonucleotide sequences used in this report can be found in Table S1. Red dashes indicate phosphate linkages and blue crosses indicate DNG linkages. 3' C3 thiol CPG is denoted by “**C_3_-SH**” and 5' thiol modifier C6 is indicated by “**HS-C_6_**”. Base colors indicate which building block was used during coupling: standard phosphoramidite is shown in red, propagating unit (**P6**) in blue, initiator unit (**I5**) in yellow and capping unit (**C3**) in green. The initiating unit enables the switch from DNA to DNG chemistry. The propagating unit is used to introduce consecutive DNG linkages, and the capping unit is used to switch from DNG to DNA chemistry.

Table S1. Oligonucleotide sequences.

| **Name** | **Sequence (5' to 3')** |
| --- | --- |
| T20-0DNG | **T-T-T-T-T-T-T-T-T-T-T-T-T-T-T-T-T-T-T-T-C_3_-SH** |
| T20-Cy5 | **Cy5-T-T-T-T-T-T-T-T-T-T-T-T-T-T-T-T-T-T-T-T-C_3_-SH** |
| T19-1DNG, 5' | **T+T-T-T-T-T-T-T-T-T-T-T-T-T-T-T-T-T-T-T-C_3_-SH** |
| T18-2DNG, 5' | **T+T+T-T-T-T-T-T-T-T-T-T-T-T-T-T-T-T-T-T-C_3_-SH** |
| T17-3DNG, 5' | **T+T+T+T-T-T-T-T-T-T-T-T-T-T-T-T-T-T-T-T-C_3_-SH** |
| T14-6DNG, 5' | **T+T+T+T+T+T+T-T-T-T-T-T-T-T-T-T-T-T-T-T- C_3_-SH** |
| T11-9DNG, 5' | **T+T+T+T+T+T+T+T+T+T-T-T-T-T-T-T-T-T-T-T- C_3_-SH** |
| T19-1DNG, 3' | **T-T-T-T-T-T-T-T-T-T-T-T-T-T-T-T-T-T-T+T- C_3_-SH** |
| T18-2DNG, 3' | **T-T-T-T-T-T-T-T-T-T-T-T-T-T-T-T-T-T+T+T- C_3_-SH** |
| T17-3DNG, 3' | **T-T-T-T-T-T-T-T-T-T-T-T-T-T-T-T-T+T+T+T- C_3_-SH** |
| A20 | **HS-C_6_-A-A-A-A-A-A-A-A-A-A-A-A-A-A-A-A-A-A-A-A** |

# 5. Characterization of DNG-DNA chimeras

## 5.1 MALDI-TOF-MS characterization of DNG-DNA chimeras

The structure of DNG-DNA chimeras was confirmed using MALDI-TOF-MS in negative mode, using DHAP as the matrix (Table S2).

Table S2. MALDI-TOF-MS characterization of DNG-DNA oligonucleotides.

| **Name** | **Calculated** | **Found** |
| --- | --- | --- |
| T20-0DNG | 6263 | 6273 |
| T19-1DNG, 5' | 6223 | 6236 |
| T18-2DNG, 5' | 6184 | 6193 |
| T17-3DNG, 5' | 6145 | 6155 |
| T14-6DNG, 5' | 6028 | 6030 |
| T19-1DNG, 3' | 6224 | 6231 |
| T18-2DNG, 3' | 6185 | 6197 |
| T17-3DNG, 3' | 6146 | 6157 |

## 5.2 Characterization of DNG-DNA chimeras using gel electrophoresis

DNG-DNA oligonucleotides were characterized using denaturing PAGE to confirm removal of failure strands and to assess the impact of charge alterations on electrophoretic mobility. Each DNG modification is expected to decrease the electrophoretic mobility of fully denatured strands of equal length as demonstrated in Figure S1A. When not fully denatured, DNG-DNA chimeras can form electrostatic assemblies leading to more condensed structures as shown below in Figure S1B. All PAGE experiments were carried by loading 0.02–0.1 OD of each strand on 15% denaturing gels (100 V, 1 h) and staining with either SYBR Safe, GelRed or Stains-All prior to imaging. For full denaturation, strands were heated at 95 °C for 5 min and loaded while hot. (Figure S1A) Self-assembly of DNG-DNA strands could be observed when heating was omitted (Figure S1B). T14-6DNG and T11-9DNG did not stain well with GelRed but could be detected using UV illumination or by staining with Stains-All. T11-9DNG could only be detected faintly on gel, we hypothesize this was due to the low solubility of this chimera in water (Figure S1C and 2A). The solubility of the crude T11-9DNG mixture can be modulated by the addition of acetic acid, SDS, 1× PBS or urea where SDS facilitated the solubilization of all species in solution, 1× PBS and urea yielded a mild improvement and acetic causes further precipitation of the oligonucleotides in solution (Figure S2B).

Figure S1. Electrophoretic mobility of DNG-DNA strands. A) Lanes 1–4 show 20mer oligonucleotides incorporating 0, 1, 2 and 3 DNG linkages at the 5' end respectively while lanes 5–8 show 20mer oligonucleotides incorporating 0, 1, 2 and 3 DNG linkages at the 3' end respectively on 15% denaturing PAGE, stained with SYBR Safe, loaded at 95 °C. B) Lanes 1–4 show 20mer oligonucleotides incorporating 0, 1, 2 and 3 DNG linkages at the 5' end respectively on 15% denaturing PAGE, stained with SYBR Safe, loaded at room temperature. C) Crude mixture following synthesis and deprotection of T11-9DNG. Image shows a preparative gel with UV illumination. Oligonucleotides with 7DNG inserts and above have low water solubility and show as very faint bands.

Figure S2. Solubility of T11-9DNG, a 20-base oligonucleotide that is nearly electroneutral. A) An image of the crude mixture for T11-9DNG in water, which yields a cloudy suspension. B) Different additives influence the solubility of the T11-9DNG crude mixture with SDS producing the best results and the addition of acetic acid yielding a thicker suspension.

## 5.3 Melting temperature by UV-visible spectroscopy

Duplexes of T20-0DNG, T19-1DNG, T18-2DNG and T17-3DNG were formed with A20 at 5 μM in 1× PBS by heating to 70 °C and slowly cooling to 4 °C in 1 h. Melts of the duplexes were acquired using UV-Vis spectroscopy, monitoring absorbance at 260 nm from 4 °C to 70 °C, at 1 °C/min (Figure S3). DNG-DNA duplexes show cooperative transitions; however, DNG modifications are destabilizing in mixed DNG-DNA duplexes, likely due to their altered internucleotide spacing. The melt experiment with T20-0DNG, T17-3DNG and T14-6DNG was performed at 2 μM in 1× PBS monitoring absorbance at 260 nm from 4 °C to 95 °C, at 1 °C/min.

Figure S3. Melting transitions of A-T duplexes with 0, 1, 2 or 3 of DNG linkages. Melts were performed with 5 μM duplexes in 1× PBS.

Table S3. Recorded melting temperature for A20 duplexes with DNA-DNG chimeras

| **DNA-DNG Chimeras** | **Melting temperature (ºC)** | **Change relative to A20:T20 (ºC)** | **Change per DNG insert (ºC)** |
| --- | --- | --- | --- |
| T20-0DNG | 53.1 | - | - |
| T19-1DNG | 51.2 | -1.9 | -1.9 |
| T18-2DNG | 50.3 | -2.8 | -1.4 |
| T17-3DNG | 49.6 | -3.5 | -1.2 |
| T14-6DNG | 43.4 | -9.7 | -1.6 |

## 5.4 Secondary structure by circular dichroism

The impact of DNG modifications on the secondary structure of the resulting DNG-DNA oligonucleotides was assessed using circular dichroism in the single-stranded (Figure S4A) and double-stranded forms (Figure S4B). DNG-DNA chimeras (5 μM in 1× PBS) were incubated at 25 °C in a 0.1 cm pathlength cuvette. CD signal was recorded from 200–400 nm at 100 nm/min with a bandwidth of 5 nm, data pitch of 1 nm and integration time of 0.5 sec. 3 accumulations were recorded for each sample. Data was smoothed using the means-movement function with a convolution width of 5. DNG-modified oligonucleotides do not deviate much from the expected spectra for unmodified T20 and T20:A20, except for the intensity of the signal, which showed a small decrease for the DNG-modified samples.

Figure S4. Circular dichroism traces for A) DNG-DNA oligonucleotides and B) A20:T20 duplexes with varying DNG inserts at 5 μM in 1× PBS buffer.

# 6. Synthesis and characterization of DNG spherical nucleic acids (SNAs)

## 6.1 Synthesis of SNAs

6.1.1 ***Salt aging method***

SNAs were synthesized based on a previous report from the group, typically at the 1–10 pmol scale.^11^ Briefly, oligonucleotides were incubated with citrate-capped 13-nm gold nanoparticles (AuNPs, obtained using previously reported protocols^1^) at a ratio of 500:1 or 1000:1 in Milli-Q filtered water with 0.1% Tween-20. It is important to note that surfactants (e.g., Tween-20) are required to obtain high yields during salt aging. In the absence of surfactants, the DNG-functionalized SNAs irreversibly adhere to tubes or glass vials during functionalization (Figure S5), consistent with other multivalent guanidinium architectures.^12^ Aliquots of sodium chloride (NaCl, 5 M solution) were added to mitigate electrostatic repulsion between the AuNPs and the oligonucleotides at 30 min intervals to reach concentrations of 0.05; 0.10; 0.20; 0.30; 0.40 up to 0.50 M. The SNAs were then incubated overnight with mixing to achieve maximal oligonucleotide density. Post-functionalization, SNAs were washed 3–5 times by pelleting (13 000 rcf, 30 min), removing the supernatant and resuspending the SNAs in Milli-Q filtered water. SNAs were stored at 4 °C until further use. Interestingly, outer surface DNG-modified SNAs were found to adhere to plastic tubes in a DNG-dependent manner as salt concentrations were increased if the surfactant was omitted, consistent with unwinding of electrostatically-stabilized secondary structures of the DNG-DNA oligonucleotides (Figure S5).

Figure S5. Extent of SNA adhesion to tubes with increasing DNG and NaCl concentrations.

6.1.2 ***Freeze-thaw method***

DNG-modified SNAs were synthesized using the freeze-thaw approach as described previously.^13^ Briefly, DNG-DNA chimeras were mixed with AuNPs at a ratio of either 300:1 or 500:1 in Milli-Q filtered water and frozen solid in liquid nitrogen or dry ice to form SNAs. SNAs were allowed to warm to room temperature, changing from a purple to a red color. Once thawed, SNAs were washed 3–5 times by pelleting (13 000 rcf, 30 min), replacing the supernatant with Milli-Q filtered water. SNAs were stored at 4 °C until further use.

6.1.3 ***pH-assisted functionalization method***

The pH-assisted functionalization method takes advantage of the protonation of bases at pH 3 to bridge the electrostatic repulsion between the oligonucleotides and the AuNPs. Functionalization was conducted according to the protocols described by Zhang *et al*.^14^ For DNG-SNAs made from polythymidyl oligonucleotides, this method proved ineffective, suggesting that the DNG inserts could not substitute for the protonatable adenine bases, which mediate the attachment to the AuNP surface.^15^

6.1.4 ***Determination of oligonucleotide loading density***

Oligonucleotide density at the surface of AuNPs was determined in two steps. First, AuNP concentrations were determined using UV-Visible spectroscopy by measuring the gold plasmon band at λ = 520 nm, using 2.76 × 10^8^ M^-1^ ⋅cm^-1^ as the extinction coefficient. Second, oligonucleotide concentrations of DNG-modified SNAs were determined by dissolving the gold core in 20 mM potassium cyanide (KCN) and then adding Quant-iT OliGreen ssDNA reagent, measuring concentrations using fluorescence measurements on a microplate reader (λ_ex_ = 480 nm, λ_em_ = 520 nm) against a calibration curve of the free oligonucleotide in 20 mM KCN. Oligonucleotide densities in this article are reported as [oligonucleotides]/[AuNPs].

## 6.2 Zeta potential and dynamic light scattering (DLS)

Zeta potential measurements consisted of 6 measurements of 30 runs performed in disposable cuvettes using 1 or 2 nM DNG-modified SNAs equilibrated for 1 h at 25 °C in Milli-Q filtered water and filtered at 0.2 μm. SNAs typically show zeta potential ≤ -30 mV. DNG-modified SNAs made either by the salt aging (Figure S6A) or the freeze-thawing approach (Figure S6B) were compared. Both methods produce SNAs with decreasing apparent surface charge as the number of DNG inserts is increased.

Dynamic light scattering consisted of 6 measurements of 13 runs performed in disposable cuvettes using 1 nM DNG-modified SNAs equilibrated for 1 h at 25 °C in Milli-Q filtered water and filtered at 0.2 μm. The expected diameter of SNAs functionalized with 20mer DNA is approximately 27 nm. DNG-modified SNAs made either by the salt aging (Figure S6C) or freeze-thawing approach (Figure S6D) were compared. In the salt aging case, DNG-modified SNAs appear to shrink as the number of DNG inserts is increased, likely due to intramolecular electrostatic interactions. SNAs obtained from the freeze-thaw approach aggregate as the number of DNGs is increased, which is likely due to the freezing portion of the process.

6.2.1 ***Effect of counterions on DNG SNAs***

Guanidiniums interact strongly with anionic groups such as carboxylates, sulfates and phosphates.^16^ To determine if the guanidinium linkages of DNG SNAs can respond to anions present in solution, DNG SNAs were incubated with sodium salts with varying counterions (chloride, sulfate and phosphate) at 0.5 M in water. Hydrodynamic diameter was determined by DLS using 6 measurements of 13 runs at 25 °C. As opposed to water and sodium chloride, sulfate and phosphate salts increase the apparent size of 6 DNG SNAs relative to unmodified SNAs. This suggests DNGs can adopt intramolecular secondary structures on the surface of SNAs, likely through electrostatic pairing of guanidinium and phosphate linkages, which are sensitive to the counterions present in solution.

Figure S6. Zeta potential of DNG SNAs containing 1-3 DNG linkages made using the A) salt aging or B) freeze-thawing approach. Hydrodynamic diameter of DNG-modified SNAs containing 1-3 DNG linkages made either by the C) salt aging or D) freeze-thawing approach.

## 6.3 Electrophoresis on agarose

DNG-modified SNAs (0.3 pmol/lane) were assessed by agarose electrophoresis using 1× Tris-glycine-SDS as the running buffer (1% agarose, 100 V, 1 h) and scanned using the densitometry setting on a Typhoon gel imager. Interestingly, freeze-thawed SNAs with external DNG modifications (5′ end, near the outer core of the SNA) show aggregation and assembly even under denaturing conditions, likely due to electrostatic interactions with the AuNPs and oligonucleotides during freezing, while the SNAs with internal DNG modifications (3′ end, near the AuNP surface) show monodisperse bands. (Figure S7) In contrast, SNAs synthesized using the salt-aging method formed monodisperse bands on gel and were colloidally stable. (Figure 2E)

Figure S7. Electrophoretic mobility of DNG-modified SNAs on 1% agarose. A) SNAs prepared using the freeze-thaw method with external DNG modifications (5′ end, outer surface of SNAs) show evidence of aggregation and assembly while SNAs with DNG modification near the nanoparticle core (3′ end, near the AuNP core of SNAs) behave like unmodified SNAs.

# 7. Impact of DNG-modifications on the cellular uptake of SNAs

## 7.1 Cell viability of DNG SNAs using the AlamarBlue assay

Oligonucleotides and SNAs containing either 0 or 3 DNG modifications were incubated with C166 cells at a final concentration of 0, 25, 50, 100, 200 or 400 nM by oligonucleotide (5 µL per sample). They were added to cells in 90 µL Opti-MEM. AlamarBlue reagent (5 µL) was added to all wells prior to incubation for 6 h at 37 ºC with 5% CO_2_. Viability was assessed by measuring fluorescence at the endpoint of the experiment (λ_ex_ = 560 nm, λ_em_ = 590 nm) on a microplate reader. 3 DNG chimeras and 3 DNG SNAs do not impact cell viability, mirroring previous findings.^2^ (Figure S8A)

Throughout ICP-MS, flow cytometry, and confocal microscopy studies, cellular toxicity was monitored by comparing cell densities in treated samples with those of untreated controls. In all cases, comparable cell counts were observed between groups, providing no evidence of cytotoxicity associated with DNG SNAs under the experimental conditions employed.

## 7.2 eGFP expression and cell viability using RT-qPCR

The effects of DNG-SNAs on cellular function and gene expression were further assessed in enhanced green fluorescent protein (eGFP)-expressing C166 cells by reverse transcription quantitative polymerase chain reaction (RT-qPCR). (Figure S8BC) C166 cells stably expressing eGFP were seeded in 12-well plates and incubated with SNAs containing 0, 1, 2, 3, or 6 DNG-modified oligonucleotides (400 nM oligonucleotide concentration) in triplicates for 4 h at 37 °C in serum-free DMEM. Subsequently, DMEM containing 30% fetal bovine serum (FBS) was added to achieve a final concentration of 10% FBS. Cells were incubated for an additional 44 h at 37 °C before RNA isolation. Total RNA was extracted using the RNeasy Mini Kit (QIAGEN) according to the manufacturer's protocol.

RNA concentration and purity were determined by UV-visible spectroscopy using a NanoDrop instrument. RT-qPCR reactions were performed in technical triplicates using qScript XLT One-Step RT-qPCR ToughMix (Quanta Biosciences) and TaqMan Gene Expression Assays for eGFP (Mr04329676_mr, FAM) and GAPDH (Mm99999915_g1, VIC) (Thermo Fisher Scientific), in 25 µL reaction volumes according to the manufacturer's instructions. Amplification was carried out on a Bio-Rad CFX384 Real-Time PCR System.

Relative eGFP expression levels were normalized to the endogenous reference gene GAPDH and expressed relative to untreated cells. Fold changes were calculated using the Pfaffl method, incorporating assay-specific amplification efficiencies.^17^ Representative results are shown in Figure S8BC. DNG SNAs did not impact eGFP expression at the concentration tested suggesting they did not negatively impact cell viability.

Figure S8. A) Cell viability of C166 cells incubated with DNG SNAs compared to free oligonucleotides as measured using the AlamarBlue assay. T17-3DNG chimeras and SNAs are non-toxic to cells in the range tested (0-400 nM) compared to the T20-0DNG unmodified control. B) eGFP expression and cell viability of eGFP-expressing C166 cells treated with DNG SNAs containing 0, 1, 2 or 3 DNGs on their outer shell (400 nM by oligonucleotide). eGFP expression and cell health is not altered for all DNG SNAs tested. C) eGFP expression and cell viability of eGFP-expressing C166 cells treated with DNG SNAs containing 0 or 6 DNGs on their outer shell (400 nM by oligonucleotide). eGFP expression and cell health is maintained for all DNG SNAs tested.

## 7.3 Cellular uptake by ICP-MS

7.3.1 ***Impact of external (5′ end) versus internal (3′ end) modifications***

HEK293 cells were incubated with 3 nM SNAs (400 nM by oligonucleotide) in duplicates in Opti-MEM at 37 °C for 5 hours in a 96-well plate, along with a cell-free control to be used as blank to account for SNA adhesion to the plate. Cells were then washed with 1× PBS and detached with 100 μL trypsin for 5 min at 37 °C. For each sample, cells were thoroughly mixed prior to transferring 80 μL into a 15 mL conical tube for digestion while 10 μL were stained with trypan blue and counted. Digestion was performed by adding equal volumes of concentrated hydrochloric acid and nitric acid for 30 min at room temperature, followed by dilution to 2% of each acid with water to a final volume of 6 mL. Samples were analyzed by ICP-MS in triplicates against a gold calibration curve built for every experiment.

7.3.2 ***Increasing DNG length near the outer surface (5′ end)***

C166 cells were incubated with 1 nM SNAs (120 nM by oligonucleotide) in triplicates in Opti-MEM (along with a cell-free blank to account for SNA adhesion to the plate) for 5 h at 37 °C. Cells were washed with 1× PBS and detached with 100 μL trypsin for 5 min at 37 °C. For each sample, cells were thoroughly mixed prior to transferring 80 μL into a 15 mL conical tube for digestion while 10 μL were stained with trypan blue and counted. Digestion was performed by adding equal volumes of concentrated hydrochloric acid and nitric acid for 30 min at room temperature, followed by dilution to 2% of each acid with water to a final volume of 6 mL. Samples were analyzed by ICP-MS in triplicates against a gold calibration curve built for every experiment.

## 7.4 Cellular uptake by confocal microscopy

7.4.1 ***SNA preparation for confocal microscopy***

Cy3- or Cy5-labeled SNAs with equal dye loading were synthesized in two steps via the salt aging approach. At first, Cy3 or Cy5-labeled oligonucleotides were incubated 500:1 with AuNPs in 0.01 M NaCl for 4 h as a large batch to ensure equal but low loading of the fluorophore. The low-density Cy3 or Cy5-labeled SNAs were washed 3 times with water by pelleting and then subdivided into small batches to which oligonucleotides with 0, 3 or 6 DNGs were added at 1000:1 with respect to the AuNPs. NaCl was added up to a final concentration of 0.5 M to form a dense layer of oligonucleotides on the surface of the AuNPs and the SNAs were left to incubate overnight. The resulting SNAs were washed 3 times by pelleting and analyzed using the Oligreen assay.

7.4.2 ***Uptake at 37 °C versus 4 °C***

HEK293 cells were incubated with DNG SNAs labeled with Cy3 in triplicates (2.5 nM by gold, 7.5 nM by dye, 225 nM by oligonucleotide) in Opti-MEM at 37 °C or 4 °C for 2 hours in an 8-well chambered coverglass. The DNG modifications were placed on the outer surface of the SNAs. Cells were washed with 1× PBS, stained with Hoechst 33342 and fixed with 2% paraformaldehyde prior to imaging using the 633 nm laser line at constant power and PMT settings (Figure S9). Cells incubated with the DNG-containing SNAs were brighter when compared to unmodified SNAs. The untreated (blank) samples show low levels of fluorescence, which were attributed to autofluorescence. Uptake a 4 ºC was limited for all SNAs suggesting active mechanisms are required for cell entry.

Figure S9. Cellular uptake of Cy3-labeled DNG SNAs containing 0, 1, 2 or 3 DNG inserts (7.5 nM by dye, 2.5 nM by gold, 225 nM by oligonucleotide) incubated for 2 hours with HEK 293 cells by confocal microscopy at 37 °C and 4 °C. Scale bar represents 20 µm.

7.4.3 ***Impact of increasing DNG inserts near the surface***

C166 cells were incubated with SNAs labeled with Cy5 in triplicates (34 nM by dye, 1 nM by gold, 120 nM by oligonucleotide) in Opti-MEM at 37 °C for 5 hours in an 8-well chambered coverglass. Cells were washed with 1× PBS and fixed with 2% paraformaldehyde prior to imaging using the 633 nm laser line at constant power and PMT settings (Figure S10). Increasing the number of DNG inserts near the surface leads to brighter signals. The untreated samples show low levels of fluorescence, which were attributed to autofluorescence.

Figure S10. Cellular uptake of Cy5-labeled DNG SNAs with 0, 3 or 6 DNG inserts near the surface (34 nM by dye, 1 nM by gold, 120 nM by oligonucleotide) incubated for 5 hours with C166 cells at 37 °C. Scale bar represents 20 µm.

## 7.5 Drug challenge by flow cytometry

Triplicate measurements in C166 cells incubated with DMEM supplemented with 10% FBS and penicillin/streptomycin at 37 °C. Cells were pre-treated with either 1× PBS, fucoidan (50 ug/ml), sodium azide (50 mM) and 2-deoxyglucose (50 mM) methyl-β-cyclodextrin (12.5 mg/mL) for 30 min according to previously reported procedures.^18^ Cy5-labeled SNAs (made using the method described in section 7.4.1) with 0, 1, 2, 3 or 6 DNG inserts at either the 5' (external modification) or 3' (internal modification) end were then added at a concentration of 3 nM by gold for 60 min at 37 °C. Cells were triple-rinsed with 1× PBS containing 0.5 mg/mL heparin at 37 °C for 5 min after incubations were completed, trypsinized and fixed with 2% paraformaldehyde for analysis by flow cytometry where the Cy5 signal was tracked in live cells for each drug treatment relative to the signal in cells treated with only 1× PBS as blanks for each given SNA.

## 7.6 Statistical analysis

Statistical analyses were performed using GraphPad Prism version 11.0.0. For cellular uptake experiments by ICP-MS, analysis of variance was performed using the Brown-Forsythe and Welch ANOVA test followed by Dunnett’s T3 multiple comparisons test, with individual variance computed for each comparison. For counterion binding experiment by dynamic light scattering and drug challenge experiments by flow cytometry, analysis of variance was conducted using a two-way ANOVA with a Bonferroni’s multiple comparisons test.

# 8. References

1	Liu, J. & Lu, Y. Preparation of aptamer-linked gold nanoparticle purple aggregates for colorimetric sensing of analytes. *Nat. Protoc.* **1**, 246-252 (2006). <https://doi.org/10.1038/nprot.2006.38>

2	Skakuj, K., Bujold, K. E. & Mirkin, C. A. Mercury-Free Automated Synthesis of Guanidinium Backbone Oligonucleotides. *J. Am. Chem. Soc.* **141**, 20171-20176 (2019). <https://doi.org/10.1021/jacs.9b09937>

3	Skakuj, K., Bujold, K. E. & Mirkin, C. A. Automated Synthesis and Purification of Guanidine-Backbone Oligonucleotides. *Curr. Protoc. Nucleic Acid Chem.* **81**, e110 (2020).

4	Challa, H. & Bruice, T. C. Deoxynucleic guanidine; synthesis and incorporation of purine nucleosides into positively charged DNG oligonucleotides. *Bioorg. Med. Chem.* **12**, 1475-1481 (2004). <https://doi.org/10.1016/j.bmc.2003.12.043>

5	Linkletter, B. A., Szabo, I. E. & Bruice, T. C. Solid-phase synthesis of oligopurine deoxynucleic guanidine (DNG) and analysis of binding with DNA oligomers. *Nucleic Acids Res.* **29**, 2370-2376 (2001). <https://doi.org/10.1093/nar/29.11.2370>

6	Eleuteri, A., Reese, C. B. & Song, Q. Synthesis of 3′,5′-dithiothymidine and related compounds. *J. Chem. Soc., Perkin Trans. 1*, 2237-2240 (1996). <https://doi.org/10.1039/P19960002237>

7	Lavandera, I., Fernández, S., Ferrero, M. & Gotor, V. First Regioselective Enzymatic Acylation of Amino Groups Applied to Pyrimidine 3‘,5‘-Diaminonucleoside Derivatives. Improved Synthesis of Pyrimidine 3‘,5‘-Diamino-2‘,3‘,5‘-trideoxynucleosides. *J. Org. Chem.* **66**, 4079-4082 (2001). <https://doi.org/10.1021/jo010048a>

8	Henningfeld, K. A., Arslan, T. & Hecht, S. M. Alteration of DNA Primary Structure by DNA Topoisomerase I. Isolation of the Covalent Topoisomerase I−DNA Binary Complex in Enzymatically Competent Form. *J. Am. Chem. Soc.* **118**, 11701-11714 (1996). <https://doi.org/10.1021/ja961788h>

9	Ruda, G. F. *et al.* Modified 5′-Trityl Nucleosides as Inhibitors of Plasmodium falciparum dUTPase. *ChemMedChem* **6**, 309-320 (2011). <https://doi.org/10.1002/cmdc.201000445>

10	Beaucage, S. L. & Caruthers, M. H. Deoxynucleoside phosphoramidites—A new class of key intermediates for deoxypolynucleotide synthesis. *Tetrahedron Lett.* **22**, 1859-1862 (1981). <https://doi.org/https://doi.org/10.1016/S0040-4039(01)90461-7>

11	Hurst, S. J., Lytton-Jean, A. K. R. & Mirkin, C. A. Maximizing DNA Loading on a Range of Gold Nanoparticle Sizes. *Anal. Chem.* **78**, 8313-8318 (2006). <https://doi.org/10.1021/ac0613582>

12	Mogaki, R., Hashim, P. K., Okuro, K. & Aida, T. Guanidinium-based “molecular glues” for modulation of biomolecular functions. *Chemical Society Reviews* **46**, 6480-6491 (2017). <https://doi.org/10.1039/C7CS00647K>

13	Liu, B. & Liu, J. Freezing Directed Construction of Bio/Nano Interfaces: Reagentless Conjugation, Denser Spherical Nucleic Acids, and Better Nanoflares. *J. Am. Chem. Soc.* **139**, 9471-9474 (2017). <https://doi.org/10.1021/jacs.7b04885>

14	Zhang, X., Servos, M. R. & Liu, J. Instantaneous and Quantitative Functionalization of Gold Nanoparticles with Thiolated DNA Using a pH-Assisted and Surfactant-Free Route. *J. Am. Chem. Soc.* **134**, 7266-7269 (2012). <https://doi.org/10.1021/ja3014055>

15	Liu, B. & Liu, J. Methods for preparing DNA-functionalized gold nanoparticles, a key reagent of bioanalytical chemistry. *Anal. Methods* **9**, 2633-2643 (2017). <https://doi.org/10.1039/C7AY00368D>

16	Best, M. D., Tobey, S. L. & Anslyn, E. V. Abiotic guanidinium containing receptors for anionic species. *Coord. Chem. Rev.* **240**, 3-15 (2003). <https://doi.org/https://doi.org/10.1016/S0010-8545(02)00256-4>

17	Pfaffl, M. W. A new mathematical model for relative quantification in real-time RT–PCR. *Nucleic Acids Res.* **29**, e45-e45 (2001). <https://doi.org/10.1093/nar/29.9.e45>

18	Choi, C. H. J., Hao, L., Narayan, S. P., Auyeung, E. & Mirkin, C. A. Mechanism for the endocytosis of spherical nucleic acid nanoparticle conjugates. *Proc. Natl. Acad. Sci. U.S.A.* **110**, 7625-7630 (2013). <https://doi.org/10.1073/pnas.1305804110>
